# Measuring Internet Slang Style in the Marketing Context: Scale Development and Validation

**DOI:** 10.3389/fpsyg.2021.751806

**Published:** 2022-01-18

**Authors:** Shixiong Liu, Yi Wu, Wu Gong

**Affiliations:** ^1^Department of Marketing, College of Management, Shenzhen University, Shenzhen, China; ^2^Asia Europe Business School, Faculty of Economics and Management, East China Normal University, Shanghai, China

**Keywords:** internet slang style, scale development, brand personality, marketing context, communication

## Abstract

As an emerging language variant, practitioners have extensively used Internet slang in advertising and other communication activities. However, its unique characteristics that differ from standard language have yet to be explored. Drawing upon interdisciplinary theories on schema and communication styles, this research makes the first attempt to conceptualize and measure these characteristics by introducing a new multi-dimensional construct, “Internet slang style,” in the marketing context. It develops and validates a new scale to measure Internet slang style along the dimensions of amiability, overtness, candor, and harshness through a series of in-depth interviews, two surveys, and one experiment with consumers. In addition, this research investigates the impact of Internet slang styles on brand personality and brand attitude. The results indicate that different Internet slang style dimensions positively correspond to different brand personality dimensions but exert no influence on brand attitude. Practically, the scale provides an easy-to-use instrument to evaluate Internet slang styles from a consumer perspective to help companies appropriately employ Internet slang in marketing communication activities.

## Introduction

The extensive usage of the Internet and social media leads to the integration of virtual and real-life (Kilicer et al., 2017). As a result, Internet slang that emerges and develops online has become part of our everyday language, and even unconsciously influences people’s psychological states and behaviors in areas such as communication and consumption (Crystal, 2006; Liu et al., 2019). For example, expressions such as “rona” or “vid” have been popular among young people to replace the formal designation “COVID-19” and to inject a sense of humor as a relief when facing the problematic current pandemic situation. Meanwhile, marketing practitioners have begun to notice the advantages of introducing Internet slang in advertisements. An example of this is Coca-Cola’s “Share a Coke” summer campaign in China, in which many popular online nicknames were selected and printed on the coke bottles [e.g., “北鼻 (Bei Bi),” a transliteration of “baby”] to generate senses of proximity and cuteness among young consumers.

Internet slang can create distinct associations in consumers’ perceptions as a unique language variant of the standard language (Crystal, 2006). These associations can be understood within the framework of language schemata, which refers to an individual’s prototypical knowledge about the language, including its underlying social and cultural meanings, typical users, contexts, and appropriate topics, as well as individuals’ beliefs about the language (Luna and Peracchio, 2005). Such understanding would help both academics and practitioners clarify the merits and demerits of Internet slang and establish criteria for selecting appropriate slang in marketing activities. For example, recent empirical research shows that Internet slang with innovative and novel characteristics can attract audience attention (Liu et al., 2019). However, no study yet has systematically investigated consumers’ perceptions about Internet slang as a whole.

Our study introduces the “Internet slang style” to address this gap, defined as consumers’ schematic perception of the characteristics conveyed by the Internet slang expressions adopted in marketing-related contexts. Drawing upon relevant theories from psychology, communication, and marketing, we aim to contribute theoretically by: (1) establishing valid conceptualization of its definition and dimensions; (2) developing an adequate scale to measure it as a multi-dimensional construct; (3) exploring its possible marketing-relevant outcomes from a consumer perspective. To do so, we first derive the definition of Internet slang style and its dimensions based on an extensive literature review. A pilot study that involves consumer interviews validates these conceptualizations. Then, following scale-development procedures, four studies are conducted to develop the scale (Studies 1–2), examine its validity and reliability (Study 3), differentiate it from the brand personality scale (Aaker, 1997), and reveal its influence on brand personality dimensions (Study 4). As far as we know, this is a very initial attempt to systematically conceptualize and empirically examine the characteristics of Internet slang, especially in the marketing domain. We also aim to contribute to practitioners by providing an easy-to-use instrument to evaluate Internet slang style.

## Conceptualizing Internet Slang Style

### Theoretical Foundation: Schema Theory

Internet slang, consisting of distinct pronunciation, word, morphology, and syntax derived from online context, is a variant of the standard language (Liu et al., 2019). The emergence of Internet slang depends on two factors: its users and the context. On the one hand, netizens, active online for a long time, create and speak Internet slang instead of the standard language to express their unique identity. On the other, compared to real communication context, online communication is more accessible, random, and secretive, constructing a different environment for the continuous evolution of Internet slang as an independent variety.

As such, we draw from schema theory to clarify the conceptual nature of Internet slang style. Schema theory describes how people recognize and understand the world by using cognitive structures to organize prior knowledge (Fiske, 1982). In this vein, schemata refer to the knowledge unit in the human mind, and an intermediary between objects and language. People organize schemata as a psychological structure network that represents shared meaning among many individuals. In the marketing context, this network allows individuals to form mental representations of ads, brands, or products, process, retrieve, and categorize information related to them, and finally facilitate decision-making (Sujan and Bettman, 1989; Halkias, 2015).

Similarly, people with direct or indirect experiences with online communication will develop a cognitive representation of Internet slang. In other words, they establish prototypical knowledge and consensual meaning of this particular language variety, forming the “style” perception of Internet slang (Jeffries and McIntyre, 2010, p. 127). As people see similarities between events or experiences during processing, and weave them into different schema categories (Schank, 1982), the “style” of Internet slang can also be further understood and measured based on the “types” of thematic meaning delivered.

### Definition of Internet Slang Style in the Marketing Context

To the best of our knowledge, the literature contains no conceptually useful definition of Internet slang style and its dimensions that can be further extended to a scale and used in business practices. Therefore, the definition of Internet slang style should be first established before scale development. To achieve this purpose, we searched extensively to identify articles on “linguistic style,” “language style,” “communication style,” and “advertising style” published in leading journals that encompass communication or linguistic topics. By carefully understanding these articles, we tried to establish an appropriate framework to introduce the conceptualization of the Internet slang style.

Recent general conceptualizations in prior literature seem to coalesce around two streams of frameworks. On the one hand, the construct “communication style” emphasizes how people communicate with others. In this vein, communication style can be “conceived to mean the way one verbally and paraverbally interacts to signal how literal meaning should be taken, interpreted, filtered, or understood (Norton, 1978, p.99).” Prior research often uses this conceptualization to evaluate different ways that service providers use to interact with customers (e.g., Webster and Sundaram, 2009; Hwang and Park, 2018). On the other hand, “linguistic style,” or “language style,” reflects the linguistic nature of a word within a sentence structure, … and the meaning of a word provided by the semantics of the word and the rest of the sentence (Hung and Guan, 2020, p. 597). Operationalization of linguistic style usually relies on LIWC, a coding dictionary that categorizes nearly 6,400 words into 89 themes (Pennebaker et al., 2015; Kovacs and Kleinbaum, 2020). Eighteen out of these 89 themes directly capture linguistic style, and can be used to predict intentions or personality traits (e.g., Lee et al., 2019; Koh et al., 2020).

General conceptualizations outlined in this section underlie divergent theoretical natures. Although linguistic styles are indeed psychologically-derived, their operationalization is based on specific linguistic features. For example, a social linguistic style is associated with the number of social intercourse-related words, such as family, employee, neighbor, and personal pronouns (Lee et al., 2019). Therefore, the linguistic style framework cannot directly describe consumers’ perceptions of Internet slang as a unique language variety. Internet slang may contain very distinct elements or rules from the standard language and generate different consumer mindsets perceptions. By contrast, the framework of “communication styles” seems more in line with the essence of our intended definition of Internet slang style (i.e., the schema theory), as it emphasizes more on people’s schematic perceptions.

Responding to the argument that language features should be examined with consideration of social meanings embedded in the applied context (Coupland, 2007; Moore, 2012), we aim to provide a conceptualization that encompasses the essence of Internet slang style specific to the marketing domain while still offering consistency with prior communication style literature. Therefore, in our research, we formally define Internet slang style as consumers’ schematic perception of the characteristics conveyed by the Internet slang expressions adopted in marketing-related contexts (e.g., in an advertisement, on the product package).

### Dimensions of Internet Slang Style in the Marketing Context

As communication style plays as the conceptual basis for Internet slang style, we reviewed all the extracted articles again, focusing on possible dimensions of this scale. Generally, communication style consists of nine dimensions: dominant, dramatic, contentious, animated, impression-leaving, relaxed, attentive, open, and friendly (Norton, 1978). However, in line with our aim of developing a parsimonious scale specific to the marketing domain, we determined whether some dimensions needed to be summarized or eliminated and whether additional components should be considered. Embedding Internet slang in the marketing context is not the same as communicating with others in everyday interactions. We, therefore, examined the unique features of Internet slang (especially those in the marketing context) to decide on possible dimensions for the conceptualization of Internet slang style.

#### Amiability

The anti-conventional nature of Internet slang determines its originality (Collot and Belmore, 1996; Liu et al., 2019). According to social information processing theory, netizens creatively employ verbal cues (e.g., foreign words, dialects, digital elements, and icons) and interaction strategies (e.g., paraphrasing, homonyms, thumbnails, reduplication, and unconventional syntax) to express and interpret social and emotional messages in online contexts (Kundi et al., 2014; Valkenburg et al., 2016). In this way, Internet slang keeps gaining novelty and freshness. It allows its users, who are young and full of entertainment spirits, to generate more favorable and attractive impressions to similar others in computer-mediated communication (Gao, 2006; Valkenburg and Peter, 2009). As a result, Internet slang may lead its users to cultivate psychological belongingness and familiarity.

These features of Internet slang correspond to both friendly and attentive communication styles proposed by Norton (1978). The friendly communication style connotes being unhostile to deep intimacy. Meanwhile, the attentive communication style reflects the extent to which a person expresses empathy and attention during interactions with other individuals (Norton, 1978). With defining characteristics such as creativity and attractiveness, Internet slang in the marketing context may lead consumers to categorize themselves and the brands as members of the online community. Consequently, consumers develop interpersonal intimacy with these brands (Postmes et al., 2000). In sum, the amiability dimension captures overall attributes that can interpret why and how consumers feel closed and attracted by Internet slang.

#### Overtness

Computer-mediated communication has been typically taken place in anonymous contexts between unacquainted partners (Valkenburg et al., 2016). Therefore, anonymity ensures that the online communication environment is relatively independent and secretive, leading people to communicate more freely and casually online than in real contexts (Turkle, 1995; Crystal, 2006). In addition, the text-based settings allow people to develop non-verbal cues (e.g., emoji, emoticons), verbal strategies (e.g., abbreviation, interjection), and rhetoric instruments (e.g., exaggeration, metaphors) to express themselves more directly (Collot and Belmore, 1996; Liebrecht et al., 2021). For example, comparing an online chat corpus and a daily interaction corpus identifies more exclamations in the first corpus (Wong et al., 2006). People tend to express more intense emotions when using Internet slang.

Such properties of Internet slang enable people to communicate because it is “self-generated in content and self-directed in emission (Castells, 2007, p.248).” These properties integrate what Norton (1978) illustrates about open, dramatic, animated, or relaxed communication styles. Specifically, an open style, in line with the self-focus of Internet slang, indicates interacting with others in a frank, sociable, unreserved, and non-secretive manner and sharing personal emotions during the communication. In addition, a relaxed style that evaluates whether the communicator feels comfortable or at ease also reflects the free and casual natures of online contexts (Norton, 1978). Meanwhile, dramatic or animated styles emphasize more on stylistic devices (e.g., exaggerations, fantasies, stories, metaphors) or non-verbal cues (e.g., eye contact, facial expressions, and gestures) that help transmit emotive expressions or highlight/understate message content (Kang and Hyun, 2012), both, therefore, can be regarded as concrete linguistic approaches that facilitate people’s self-expression online. Collectively, the overtness dimension reflects how Internet slang can be used to express oneself and show autonomy, with signature features such as freedom, vitality, and passion.

#### Candor

Computer-mediated communication is associated with physical features such as time and space constraints and text-based (Valkenburg et al., 2016). Therefore, many informal expressions emerge and result in a popular and funny way of communication that mimics spoken language (Liu et al., 2019; Liebrecht et al., 2021). For example, audible elements can be employed as paralinguistic approaches in computer-mediated communication using repeated punctuation (“*!!!*,” “*??!!*”) and sound mimicking (“*soooo*”), while informal verbal cues include contractions (“*LOL*,” “*OMG*”) and interjections (“*wow*,” “*haha*”) (Tagliamonte, 2016; Liebrecht et al., 2021). Overall, these newly-developed unique vocabulary and phrases deliver intense meaning in a relatively shortened and straight forward manner (Wong et al., 2006; Tagliamonte, 2016), fostering another dimension of Internet slang style—condor, which refers to the conciseness, simplicity, and efficiency manifested in the usage of Internet slang during communication to others (Collot and Belmore, 1996; Crystal, 2006).

However, after careful examination, no specific communication style proposed by Norton (1978) is identical to such a candor dimension, except for the impression-leaving style. It reflects the extent to which a communicator is strongly memorable (Kang and Hyun, 2012), partially supporting what is suggested in the candor dimension. To establish a lasting impression, communicators should use unique appearance, visual stimuli, or special comments to create a meaningful memory for consumers (Hwang and Park, 2018), just as unique expressions are dynamically generated to communicate efficiently in the online context.

#### Harshness

The anonymity associated with the Internet can also lead to inhibited behaviors in online communication, such as flaming (Valkenburg et al., 2016). For example, empirical research shows that people may have the disposition to behave in an uninhibited and non-conforming manner and ignore status differences in computer-mediated communication due to the decreased social context cues of the Internet environment (Sproull and Kiesler, 1986).

Two remaining communication styles proposed by Norton (1978) correspond to these characteristics: a dominant style refers to taking charge of social interactions, while a contentious style emerges as a covariate of dominant style and denotes an argumentative and disputable way of interaction (Norton, 1978; Kang and Hyun, 2012). Remarkably, the contentious style sometimes entails negative components (Norton, 1978). Extending to our research settings, Internet slang may consist of ironic and sarcastic expressions that possess a dominant or contentious style in people’s perception. We merge these two specific styles into the harshness dimension.

The four resulting dimensions of Internet slang style are amiability, overtness, candor, and harshness. We refer to them as “ISS dimensions” in the following discussion. We propose that these four dimensions are the most prominent perceptions that Internet slang leaves to consumers. We conducted a pilot study to examine whether consumers also conceive of Internet slang style along these theoretically-driven dimensions. Six consumers were interviewed (aged 20–30 years with balanced demographics and Internet engagement, see Appendix 1 for demographic information). We asked them to indicate their opinions toward Internet slang in daily consumption (both online and offline). The interview started open-ended, but questions regarding Internet slang characteristics were also introduced later on. All interviews were recorded and then transcribed. Then, we analyzed the interviews and found considerable overlap between consumers’ responses regarding their perceptions of Internet slang and our four dimensions. Table 1 gives examples of quotations.

**TABLE 1:** Consumer quotations for the four ISS dimensions.

Dimension	Consumer quotations
Amiability dimension	• “I think Internet slang’s original and fashionable associations will transfer to my products.” • “Probably the slang I used is relatively euphemistic and mild, containing emotional feelings such as fun.” • “Usage of Internet slang makes people in the conversation feel warm.” • “I feel intimate, familiar, and adorable about Internet slang.”
Overtness dimension	• “Slangs represent characteristics of young people, such as coolness, confidence and without upper-limit.” • “Internet slangs generally possess characteristics like humor and popularity.” • “Internet slang can make communications livelier.”
Candor dimension	• “Internet slang is more comprehensible, more concise, and easier to remember.” • “Internet slang can precisely express our intended meanings. “ • “The meanings of these words and phrases are clear.” • “Internet slang helps us communicate as much information as possible in a short time.” • “By using Internet slang, only a few words will express the meaning clearly. However, using common language requires many words to type in.”
Harshness dimension	• “Some Internet slang expressions are cynical.” • “(Some Internet slangs) involve derogation, irony, and arrogation.” • “It’s informal and non-deferential features flaw internet slangs. Thus, we should be cautious about using Internet slang.” • “(Internet slang) engages sarcasm and irony toward real life.”

*The interviews were conducted in Chinese and were translated.*

## Methods and Results

Four studies were conducted to measure, validate, and establish the ISS construct and its predictivity. Study 1 focuses on item generation and purification. In Study 2, we develop and validate the ISS scale. Study 3 further validates the ISS scale and its dimensionality, while Study 4 examines ISS’s predictive power for brand personality dimensions.

### Study 1: Item Generation and Purification

The item generation procedure followed Churchill’s (1979) scale development. To generate the initial pool of 156 statements, three sources were used: (1) the literature on linguistic style, geographical dialects, and accent characteristics (Cheyne, 1970; Edwards and Jacobsen, 1987; Tsalikis et al., 1992), including Chinese classics; (2) in-depth interviews with consumers to elicit descriptions regarding ISS; and (3) additional search for online resources and Chinese dictionaries.

To eliminate redundancy, five marketing or economics graduate students were recruited to judge the statements after presenting the ISS concept and example. The appropriateness in the marketing context was explicitly listed as a critical evaluative standard. This process reduced the initial 156 statements to only 55 items. Afterward, the authors invited three marketing professors and one linguistic professor to omit to ensure the authority and rationality of the results and further reduced the statements to a manageable set of 35 items.

### Study 2: Initial Identification of Dimensionality

To identify a comprehensive and representative set of Internet slang, the authors used the ‘‘Network Buzzwords’’ database, a Chinese official website^1^ collecting Internet slang sentences voted on for popularity by Internet users. The collection period was from January 12, 2014, to December 24, 2014. The authors gathered 1,241 sentences, to which 280 additional sentences were supplemented after thoroughly reviewing a professional Chinese language journal [“*Yao Wen Jiao Zi* (咬文嚼字)”] and exploring rankings published by a Chinese online search engine (“Baidu”).

A set of 350 Internet slang sentences was randomly selected from the sentence pool, which was constructed according to the definitive standards in the pilot study and Study 1. Then, these sentences were distributed into 44 groups^2^, and 44 versions of the questionnaire were designed to limit fatigue and boredom. Specifically, each participant was randomly assigned to one version of the questionnaire and indicated their agreement with how each Internet slang sentence reflected the original 35 items generated from Study 1. All 35 items were rated on a five-point Likert scale anchored by 1 (*Strongly disagree*) and 5 (*Strongly agree*).

Participants were approached at airports, railway stations, or a university in a major city in southern China. A final set of 443 complete questionnaires was returned and reserved (valid rate = 76.3%, 47% male, 528 adults participated). Of the valid responses, 50.8% of participants were aged between 18 and 22 years, and 54.6% spent 2–4 h online daily.

Because our objective was to identify the dimensionality of ISS from consumers’ perspective and not individual variations in evaluations of each sentence, the average scores of each sentence for each item were computed across participants (each sentence evaluated by an average of 10 participants). The resulting data consisted of 350 cases, each reflecting an Internet slang sentence with average scores on 35 different items.

Iterated-principal-component exploratory factor analyses (EFAs) with direct-oblimin-rotation were conducted. First, item-to-total correlations less than 0.4 were eliminated (resulting in one item being deleted). Then, 22 items were removed individually based on the rotated component matrix and the expected interpretable meanings brought to the structure, as their loading coefficients exceeded 0.4 in more than one factor. The remaining items entered the final factor analysis, presenting a four-factor solution. The Kaiser–Meyer–Olkin value of 0.81 (Bartlett’s test of sphericity: χ^2^ = 1789.55, *p* < 0.001) and the eigenvalues greater than one both indicated that the analysis was appropriate for the data. The final set of 15 items accounted for 62.37% of the variance, and the Cronbach’s α was 0.89, within the guidelines for scale development (Nunnally, 1978). The scree plot showed a significant dip, confirming the rationality of this four-factor solution. The four factors were labeled amiability (five items, α = 0.84), overtness (five items, α = 0.78), candor (three items, α = 0.63), and harshness (two items, α = 0.66). A summary of the factor analysis is shown in Table 2.

**TABLE 2:** Four dimensions of ISS.

Name	Dimensions	Variance explained	Eigenvalue	Statement with highest item-to-total correlations
Amiability	1	30.22%	4.53	Fresh, beautiful, euphemistic, adorable, original
Overtness	2	13.64%	2.05	Lively, pure, passionate, free, popular
Candor	3	10.37%	1.56	Concise, self-mocking, forthright
Harshness	4	8.14%	1.22	Sharp, rough

### Study 3: Dimensionality Confirmation

To confirm whether the four-dimension solution was the general structure for ISS, additional research was conducted to collect data from a second independent sample of subjects to examine the structure *via* a series of confirmatory factor analyses. Similar to the procedure from Study 2, a total of 415 questionnaires were sent to participants with a similar demographic profile.

Finally, 350 useable responses (a response rate of 84.3%) formed the basis for the iterated confirmatory factor analysis models (Table 3): (a) the null model; (b) a one-factor model (all items were loaded on a single factor); (c) a two-factor correlated model (amiability and overtness items were loaded on the same factor, while the remaining items loaded on the other; (d) a four-factor uncorrelated model with items loaded on their respective hypothesized factors; (e) a four-factor correlated model with the same structure as model (d); and (f) a four-factor model with one second-order factor.

**TABLE 3:** Study 3 model comparison.

Model	χ2	df	χ2/df	CFI	GFI	IFI	RMSEA
(a) Null	1,749.60	105	NA	NA	NA	NA	NA
(b) 1-Factor	781.49	89	8.78	0.58	0.73	0.58	0.15
(c) 2-Factor correlated	753.92	88	8.57	0.61	0.73	0.61	0.15
(d) 4-Factor uncorrelated	1,243.72	97	12.82	0.64	0.66	0.64	0.18
(e) 4-Factor correlated	243.20	83	2.93	0.90	0.92	0.90	0.07
(f) 4-Factor second-order	244.10	85	2.87	0.90	0.92	0.90	0.07

*df, degrees of freedom; CFI, comparative fit index; GFI, goodness of fit index; IFI, incremental fit index; RMSEA, root mean square error of approximation.*

*NA suggests that the corresponding estimates do not exist.*

Table 3 shows that, compared with the fit results of models (a)–(d), models (e) and (f) presented superior fit results (Kelloway, 1998). Model (e) was constructed as a correlated four-factor model (χ^2^/*df* = 2.93, CFI = 0.90, GFI = 0.92, IFI = 0.90, RMSEA = 0.07). Model (f) was to confirm these four factors constituted the higher-order construct ISS (χ^2^/*df* = 2.87, CFI = 0.90, GFI = 0.92, IFI = 0.90, RMSEA = 0.07). Closer examination revealed identical results from these two models. First, nearly every indicator’s *t* value was statistically significant (*p* < 0.01), and almost all the coefficients exceeded 0.50 (Table 4). Furthermore, the estimates of Cronbach’s α for the ISS four factors based on the second independent sample were similar to those of Study 2: amiability α = 0.83, overtness α = 0.78, candor α = 0.64, and harshness α = 0.74. Finally, composite reliability and AVE indices were computed to justify the scale’s convergent and discriminant validity (Fornell and Larcker, 1981; Table 5). Composite reliability was higher than 0.60, and AVE did not fall below the corresponding pairwise squared correlation coefficients. In general, results supported the stability of the four-factor structure for the scale and suggested good psychometric properties.

**TABLE 4:** Coefficients of the first- and second- order four-factor CFA models in study 3.

Paths	First order CFA result		Second order CFA result	
	Estimates	*t*-values	Estimates	*t*-values
Amiability → Euphemistic	0.67	NA	0.67	NA
Amiability → Beautiful	0.71	10.82	0.71	10.81
Amiability → Fresh	0.60	9.38	0.60	9.38
Amiability → Adorable	0.77	11.43	0.76	11.43
Amiability → Original	0.72	10.95	0.72	10.95
Overtness → Free	0.58	NA	0.58	NA
Overtness → Lively	0.81	10.37	0.81	10.37
Overtness → Pure	0.79	10.25	0.79	10.26
Overtness → Passionate	0.71	9.64	0.71	9.65
Overtness → Popular	0.36	5.80	0.36	5.80
Candor → Self-mocking	0.61	NA	NA	NA
Candor → Concise	0.55	6.74	0.55	6.71
Candor → Forthright	0.64	7.04	0.65	7.03
Harshness → Sharp	0.68	NA	0.68	NA
Harshness → Rough	0.86	6.74	0.86	6.74
Overall → Amiability	NA	NA	0.72	Fixed
Overall → Overtness	NA	NA	0.49	4.76
Overall → Candor	NA	NA	0.68	5.04
Overall → Harshness	NA	NA	0.57	4.65

*Overall refers to the second-order construct Internet slang style. NA suggests that the corresponding estimates do not exist.*

**TABLE 5:** Tests for convergence and discriminant validity in study 3.

Dimensions	First-level CFA results				Second-level CFA results				
	Amiability	Overtness	Condor	Harshness	Amiability	Overtness	Condor	Harshness	Overall
Amiability	0.48 (0.82)	0.09	0.09	0.10	0.48 (0.82)	0.09	0.09	0.10	NA
Overtness	0.31	0.45 (0.79)	0.07	0.07	0.31	0.45 (0.79)	0.07	0.07	NA
Condor	0.30	0.27	0.36 (0.63)	0.09	0.30	0.27	0.36 (0.63)	0.09	NA
Harshness	0.32	0.26	0.30	0.61 (0.75)	0.32	0.26	0.30	0.61 (0.75)	NA
Overall	0.76	0.74	0.61	0.57	0.76	0.74	0.61	0.57	0.39 (0.71)

*The first number of the diagonal elements represents AVE; the number in parentheses refers to composite reliability.*

*The below-diagonal elements refer to correlations between dimensions; the off-diagonal elements are corresponding squared correlation.*

*NA suggests that the corresponding estimates do not exist.*

### Study 4: Predictive Validity Assessment

Study 4 was an experiment to test how different ISS dimensions induced by slogans help build corresponding brand personality dimensions in advertisements with the same product. Besides, we also wanted to provide further evidence to differentiate it from brand personality in this study.

ISS theoretically differs from brand personality, whose formal definition is “the set of human characteristics associated with a brand” (Aaker, 1997). Brand personality reflects consumers’ generalizable impressions of the brands (Aaker et al., 2004) and is helpful for companies to establish deeper consumer-brand relationships and favorable brand attitudes (Fournier, 1998). Prior research reveals that even subtle marketing cues can influence consumers’ brand personality perception, including visual symmetry (Bajaj and Bond, 2018), haptic product attributes (Ranaweera et al., 2021), and disclosure of the brand’s corporate social responsibility activities (Tarabashkina et al., 2020). More importantly, extending prior findings of linguistic reflexes of personality (Mairesse and Walker, 2011; Kovacs and Kleinbaum, 2020), we propose that brand personality can also be shaped by the specific language features adopted in the advertising. For example, using metaphors in marketing communications leads consumers to perceive the products as more innovative and less socially responsible (Luffarelli et al., 2021).

To examine the effectiveness of ISS, we adopted brand personality as the key criterion variable. For operationalization, we used the brand personality scale developed by Aaker (1997), broadly validated and generalized cross-culturally and widely applied to academic and practical settings, despite slight changes in some countries (Aaker et al., 2001). Precisely, the scale consists of five trait dimensions: sincerity, competence, excitement, ruggedness, and sophistication. Our theoretical model highlighted the trait dimensions of sincerity and competence, which constantly emerge as parts of brand personality in both eastern and western cultures (Aaker et al., 2001). Accordingly, to further understand how brand personality would dictate the kind of language used in advertisements, two ISS dimensions with relatively orthogonal meanings, amiability, and harshness, were selected in advance.

The trait of sincerity captures the extent to which consumers characterize a brand with adjectives such as “warmth,” “cheerful,” and “genuineness,” while the competence dimension is composed of efficient, successful, and confident impressions (Aaker, 1997; Aaker et al., 2001). On the one hand, consumers often form the perception of brand sincerity when a proximal psychological distance is elicited (Hu and Shi, 2020) or when a sense of social belongingness is boosted (Chang et al., 2019). In this vein, an amiable Internet slang slogan generates an atmosphere with original, pleasing, and adorable properties, allowing consumers to feel psychologically close to the brand to establish a sincere brand perception. On the other hand, a harsh slogan would convey a sense of straightforwardness and roughness, which easily leads consumers to induce associations of confidence and capability from the brand to embed in the advertisement, thereby building perceptions of brand competence (Chen, 2021). Therefore, we formally hypothesized that:

*H_1_:* Styles of Internet slang sentences significantly affect brand personality dimensions.

*H_1*a*_:* An amiable (vs. harsh) statement as a slogan causes consumers to perceive a sincere (vs. competent) brand personality.

*H_1*b*_:* A harsh (vs. amiable) statement as a slogan causes consumers to perceive a competent (vs. sincere) brand personality.

#### Design, Procedure, and Sample

In a pretest (*n* = 39, 85.3% female), participants evaluated dimensions of ISS on several network buzzwords. Among them, *Mo Mo Da* (么么哒, slogan A) scored significantly higher on the amiability dimension than did “Even today you are standoffish and indifferent to me, I will be the one out of your league sooner or later” (今天你对我爱理不理, 明天我让你高攀不起, slogan B) [*M*_A_ = 4.31, SD = 1.19; *M*_B_ = 3.15, SD = 1.04; *t*(76) = 4.56, *p* < 0.001], whereas slogan B scored significantly higher on the harshness dimension than did slogan A [*M*_A_ = 2.30, SD = 1.21; *M*_B_ = 4.53, SD = 1.44; *t*(76) = 7.42, *p* < 0.001]. Slogans A and B were not significantly different on the dimensions of overtness and candor [overtness: *M*_A_ = 4.07, SD = 1.18; *M*_B_ = 4.06, SD = 0.97; *t*(76) = 0.04, *n.s.*; candor: *M*_A_ = 4.10, SD = 1.06; *M*_B_ = 3.98, SD = 1.045; *t*(76) = 0.50, *n.s.*]. The results demonstrated that these two slogans were ideal stimuli in the follow-up experiment.

A single-factor between-group design (ISS: amiable vs. harsh) was adopted. Specifically, the experiment was administered online, and 101 Internet users (76.4% female, *M*_age_ = 24.3 years, 93.1% spent more than 2 h online every day) were randomly assigned to one condition. In the beginning, participants watched a randomly assigned advertisement for the wallet produced by a fictional brand AROX. The slogan used in the advertisements was either the amiable phrase or the harsh phrase identified in our pretest and adjusted to ensure equivalent sentence length. Except for the slogan, the two advertisements (including product pictures and layout) were the same. Then, participants were instructed to indicate their perceptions toward the wallet itself by using two items (i.e., adorable, upscale). Afterward, participants rated the slogan on two dimensions of the ISS scale (amiability α = 0.77, and harshness α = 0.77) and assessed brand personality (“What personality do you think Brand AROX possesses?” Specifically, sincerity (α = 0.74; “wholesome,” “cheerful,” “warm”) and competence (α = 0.82; “successful,” “efficient,” “determination”) were each measured by three items. Four items also measured participants’ attitudes toward the brand AROX (α = 0.85; “I like the brand,” “I’d like to buy the brand,” “It is more possible for me to buy the brand,” and “I think the brand’s quality is good”). All items were measured on a seven-point scale (1 = *Strongly disagree*, 7 = *Strongly agree*). Finally, participants answered several demographic questions and were then debriefed.

#### Results

Consistent with our pretest, participants who watched advertisements that contained slogan A rated the advertisement as more amiable and less harsh than did those who watched advertisements with slogan B [amiability: *M*_A_ = 4.43, SD = 1.00; *M*_B_ = 3.24, SD = 1.24; *t*(99) = 5.33, *p* < 0.001; harshness: *M*_A_ = 2.22, SD = 1.18; *M*_B_ = 4.57, SD = 1.28; *t*(99) = −9.58, *p* < 0.001]. Results confirmed that different dimensions of ISS were successfully manipulated. Groups exhibited no statistical difference in the perception of the product as adorable [*M*_A_ = 3.52, SD = 1.63; *M*_B_ = 3.51, SD = 1.63; *t*(99) = 0.03, *n.s.*] and upscale [*M*_A_ = 4.24, SD = 1.32; *M*_B_ = 4.31, SD = 1.61; *t*(99) = −0.25, *n.s.*] before watching the advertisements; this indicated that participants in different groups had similar perceptions of the wallet itself.

According to our hypothesis, different styles of slogan embedded in advertisements would exert different types of brands. The independent sample *t* test (ISS dimension: amiable vs. harsh) on sincerity revealed the predicted pattern [*t*(99) = 2.36, *p* < 0.05]. Specifically, the amiable slogan made participants feel the brand was more sincere than the harsh one did (*M*_A_ = 4.40, SD = 1.23; *M*_B_ = 3.82, SD = 1.25). By contrast, the harsh slogan made participants feel the brand was more competent than the amiable one did [*M*_A_ = 3.47, SD = 1.21; *M*_B_ = 4.29, SD = 1.30; *t*(99) = −3.31, *p* < 0.001]. Additionally, neither condition elicited distinctively favorable evaluations toward the brand [*M*_A_ = 3.52, SD = 1.21; *M*_B_ = 3.67, SD = 1.20; *t*(99) = −0.63, n.s.].

To summarize, the results from Study 4 verified the predictive validity of the ISS scale by showing that online slogans adopted in advertising slogans would change consumers’ perceptions of brand image (i.e., brand personality). Meanwhile, consumers’ brand evaluation was not influenced.

## Discussion

### Theoretical Contribution

Although Internet slang is recognized as a novel approach for corporations to conduct marketing practices, some gaps still exist in understanding its characteristics distinct from those of the standard language. In closing these gaps, this research attempts to link three literature streams–the communication literature, psychology literature, and marketing literature–by introducing a new construct that evaluates the unique characteristic associated with Internet slang as an emerging language variant. In this vein, we make three significant theoretical contributions.

First, some previous studies on Internet slang have focused only on one particular feature (e.g., humor or novelty) (e.g., Liu et al., 2019). We develop a theoretically well-grounded and comprehensive conceptualization of the Internet slang style as a multi-dimensional construct based on the schema theory and the communication style literature. Specifically, Internet slang style comprises the four dimensions of amiability, overtness, candor, and harshness. Accordingly, a scale of Internet slang style is constructed to provide researchers with an instrument for contextualized Internet slang research in the marketing domain. Investigators can select particular dimensions or comprehensively use our scale in their study. As such, the understanding of Internet slang and its influence in the marketing context can be deepened, and the results can be comparable.

Second, in addition to empirical results that consistently show high convergent and discriminant validity of the ISS scale (studies 1–3), we demonstrate that our scale differs from brand personality and brand attitudes under experimental conditions (Study 4). Specifically, we empirically show that Internet slang style dimensions impact consumers’ perception of brand personalities, but exert no influence on their brand attitudes. These results provide support for recent conceptual propositions that brand personality can be constructed through the linguistic identity of a brand (Carnevale et al., 2017) and extend the classic concept of “linguistic styles as the individual difference (Pennebaker and King, 1999)” to a marketing context. Meanwhile, these empirical findings also respond to the call of emphasis on exploring whether social media context (i.e., Internet slang) would infuse a collectively-derived meaning into brands (i.e., brand personality) (Carnevale et al., 2017).

Third, this research explores an interdisciplinary topic by examining Internet slang and its influence on marketing communications. In such a field that bridges linguistics and marketing theories, most research focuses on the code-switching effect (e.g., Lin and Wang, 2016; Ahn et al., 2017), or impacts of concrete linguistic elements (e.g., pronouns, phonetics, rhyme) (e.g., Hung and Guan, 2020; Liebrecht et al., 2021). Few studies have examined Internet slang individually as an essential phenomenon. This paper extends this research field by establishing a typology of the prominent perceptual characteristics manifested in Internet slang as a unique language variety.

### Managerial Implication

Marketing practitioners already acknowledge the importance of Internet slang by extensively integrating popular slags into their advertisements. Therefore, our research helps managers to gain insights into applying Internet slang in four ways. First, a refined definition of Internet slang style provides practitioners an objective recognition and shared understanding of the unique but common perceptual characteristics that Internet slang would generate among consumers. This would help establish an essential foundation for managers who consider employing Internet slang in marketing communications, and avoid possible risks of subjective judgment of Internet slang.

Second, we conceptualize and operationalize ISS as comprising four dimensions (i.e., amiability, overtness, candor, and harshness). This expanded conceptualization of the Internet slang style shows that solely associating Internet slang with separated features such as novelty, youth, or interestingness is inadequate to marketing practitioners (e.g., Crystal, 2006; Liu et al., 2019). Study 2 reveals that although amiability contributed to the highest variance, overtness, candor, and harshness each accounted for approximately 10% of the variance in the EFA. Therefore, companies should consider all four dimensions and their possible influences in their decisions to use Internet slang in their marketing communications. For instance, when adopting concise or efficient Internet slang in an advertising context, companies should realize that the slang statement might convey sharpness and even imply non-deference.

Third, we provide an easy-to-apply scale that consists of only 15 clear items for companies to measure Internet slang styles. As such, this instrument enables practitioners to predict consumers’ conception of particular Internet slang statements before launching an advertisement or promotional activity. In addition, with a large body of Internet slang available for marketing use, practitioners can establish a corpus in which all the Internet slang phrases or sentences are categorized by specific ISS dimensions that are evaluated and determined in large-scale consumer surveys beforehand. Such a corpus would allow companies to choose appropriate slang embedded in the advertisements to activate particular associations among consumers.

Finally, we find evidence that different Internet slang style dimensions correspond to differential brand personalities (Study 4). As such, Internet slang could serve as a means for building a particular brand personality. Therefore, companies should align their use of Internet slang statements with branding decisions. Strategic promotional activities that include Internet slang should not violate the brand’s predetermined personality. In turn, when launching a new brand or new product in the market, marketing managers can also take advantage of the readily available associations of certain popular Internet slang statements to construct a particular brand personality in a much easier manner.

### Limitations and Future Research

This research still contains some limitations, which also suggest avenues for future research. First, the external validity of the ISS scale and related findings might be limited because all the studies were conducted in China. Although our conceptualization encompasses general theories of psychology, communication, and marketing that involve no country-specific factors, replication studies in other countries are required in the future. Second, Internet slang is context-specific (which is ignored in the current paper). Therefore, it would be helpful to account for the interactional roles of product categories, launching platforms, and target audiences when examining the downstream effect of ISS. Third, the relationships between ISS dimensions and brand personality dimensions require more theoretical development and clarification. For example, what would the impact of overtness and candor be on brand personality dimensions? Would amiability and harshness still influence consumers’ perception of the other two brand personality dimensions? Finally, the nomological validity of the ISS scale should be further tested. This research only identifies brand personality as a possible outcome, but finds no relationship between ISS and brand attitude. Future research should reveal relationships between ISS dimensions and other consequential variables (e.g., word-of-mouth intentions) and try to identify possible antecedents that help build different ISS dimensions.

## Data Availability Statement

The raw data supporting the conclusions of this article will be made available by the authors, without undue reservation.

## Ethics Statement

Ethical review and approval was not required for the study on human participants in accordance with the local legislation and institutional requirements. Written informed consent for participation was not required for this study in accordance with the national legislation and the institutional requirements.

## Author Contributions

SL and YW conceived of the presented idea and developed the theory and research model. SL and WG collected data and performed the analysis. YW wrote the manuscript. All authors contributed to the article and approved the submitted version.

## Conflict of Interest

The authors declare that the research was conducted in the absence of any commercial or financial relationships that could be construed as a potential conflict of interest.

## Publisher’s Note

All claims expressed in this article are solely those of the authors and do not necessarily represent those of their affiliated organizations, or those of the publisher, the editors and the reviewers. Any product that may be evaluated in this article, or claim that may be made by its manufacturer, is not guaranteed or endorsed by the publisher.

## Footnotes

1 Source: https://www.wllxy.net/

2 One group contained only six different sentences; the other 43 groups had eight.

## Appendix

**TABLE A1:** Demographic statistics of pilot study participants.

No.	Gender	Age	Education level	Occupation	Daily online duration
P1	Male	20–25	Master	Student	5–8 h
P2	Male	20–25	Master	Student	3–5 h
P3	Female	25–30	Undergraduate	Company employee	8–10 h
P4	Male	25–30	Undergraduate	Self-owned electronic business	More than 12 h
P5	Female	20–25	Junior College	Company employee	8–10 h
P6	Female	25–30	Doctoral	Student	5–8 h

## References

[B1] Aaker J. L. (1997). Dimensions of brand personality. *J. Mark. Res.* 34 347–356.

[B2] Aaker J. L. Benet-Martinez V. Garolera J. (2001). Consumption symbols as carriers of culture: a study of Japanese and Spanish brand personality constructs. *J. Pers. Soc. Psychol.* 81 492–508. 10.1037/0022-3514.81.3.492 11554649

[B3] Aaker J. L. Fournier S. Brasel S. A. (2004). When good brands do bad. *J. Consum. Res.* 31 1–16. 10.1086/383419

[B4] Ahn J. La Ferle C. Lee D. (2017). Language and advertising effectiveness: code-switching in the Korean marketplace. *Int. J. Advert.* 36 477–495.

[B5] Bajaj A. Bond S. D. (2018). Beyond beauty: design symmetry and brand personality. *J. Consum. Psychol.* 28 77–98. 10.1002/jcpy.1009

[B6] Carnevale M. Luna D. Lerman D. (2017). Brand linguistics: a theory-driven framework for the study of language in branding. *Int. J. Res. Mark.* 34 572–591.

[B7] Castells M. (2007). Communication, power and counter-power in the network society. *Int. J. Commun.* 1 238–266.

[B8] Chang Y. Li Y. Yan J. Kumar V. (2019). Getting more likes: the impact of narrative person and brand image on customer–brand interactions. *J. Acad. Mark. Sci.* 47 1027–1045. 10.1007/s11747-019-00632-2

[B9] Chen Y. S. A. (2021). Does outward appearance appeal to the inward mind? The impact of packaging finishes on brand impressions and the subsequent behavior of consumer. *J. Product Brand Manag.* 30 768–778. 10.1108/jpbm-07-2019-2466

[B10] Cheyne W. M. (1970). Stereotyped reactions to speakers with Scottish and English regional accents. *Br. J. Soc. Clin. Psychol.* 9 77–79. 10.1111/j.2044-8260.1970.tb00642.x

[B11] Churchill G. A. (1979). A paradigm for developing better measures of marketing constructs. *J. Mark. Res.* 16 64–73. 10.2307/3150876

[B12] Collot M. Belmore N. (1996). *Electronic Language: a New Variety of English.* Amsterdam: John Benjamins Publishing Company.

[B13] Coupland N. (2007). *Style: Language, Variation and Identity.* Cambridge: Cambridge University Press.

[B14] Crystal D. (2006). *Language and the Internet.* Cambridge: Cambridge University Press.

[B15] Edwards J. Jacobsen M. (1987). Standard and regional standard speech: distinctions and similarities. *Lang. Soc.* 16 369–379. 10.1017/s0047404500012458

[B16] Fiske S. T. (1982). “Schema-triggered effect: applications to social perception,” in *Proceedings of the Affect and Cognition: 17th Annual Carnegie Mellon Symposium on Cognition* (Hillsdale: Lawrence Erlbaum), 55–78.

[B17] Fornell C. Larcker D. F. (1981). Evaluating structural equation models with unobservable variables and measurement error. *J. Market. Res.* 18, 39–50. 10.1177/002224378101800104

[B18] Fournier S. (1998). Consumers and their brands: developing relationship theory in consumer research. *J. Cons. Res.* 24, 343–373. 10.1086/209515

[B19] Gao L. W. (2006). Language contact and convergence in computer-mediated communication. *World Englishes* 25 299–308. 10.1111/j.0083-2919.2006.00466.x

[B20] Halkias G. (2015). Mental representation of brands: a schema-based approach to consumers’ organization of market knowledge. *J. Product Brand Manag.* 24 438–448. 10.1108/jpbm-02-2015-0818

[B21] Hu T. Shi B. (2020). More proximal, more willing to purchase: the mechanism for variability in consumers’ purchase intention toward sincere vs. exciting brands. *Front. Psychol.* 11:1258. 10.3389/fpsyg.2020.01258 32670148 PMC7330119

[B22] Hung Y. C. Guan C. (2020). Winning box office with the right movie synopsis. *Eur. J. Mark.* 54 594–614.

[B23] Hwang J. Park S. (2018). An exploratory study of how casino dealer communication styles lead to player satisfaction. *J. Travel Tour. Mark.* 35 1246–1260. 10.1080/10548408.2018.1488648

[B24] Jeffries L. McIntyre D. (2010). *Stylistics.* Cambridge: Cambridge University Press.

[B25] Kang J. Hyun S. S. (2012). Effective communication styles for the customer-oriented service employee: inducing dedicational behaviors in luxury restaurant patrons. *Int. J. Hosp. Manag.* 31 772–785.

[B26] Kelloway E. K. (1998). *Using LISREL for Structural Equation Modeling: a Researcher’s Guide.* Thousand Oaks, CA: Sage Publications.

[B27] Kilicer K. Coklar A. N. Ozeke V. (2017). Cyber human values scale (i-value): the study of development, validity, and reliability. *Internet Res.* 27 1255–1274. 10.1108/intr-10-2016-0290

[B28] Koh Y. Lee M. Kim J. Yang Y. Y. (2020). Successful restaurant crowdfunding: the role of linguistic style. *Int. J. Contemp. Hosp. Manag.* 32 3051–3066.

[B29] Kovacs B. Kleinbaum A. M. (2020). Language-style similarity and social networks. *Psychol. Sci.* 31 202–213. 10.1177/0956797619894557 31877069

[B30] Kundi F. M. Shakeel A. Aurangzeb K. Asghar M. Z. (2014). Detection and scoring of internet slangs for sentiment analysis using SentiWordNet. *Life Sci. J.* 11 66–72.

[B31] Lee C. H. Bian Y. Karaouzene R. Suleiman N. (2019). Examining the role of narratives in civic crowdfunding: linguistic style and message substance. *Ind. Manag. Data Syst.* 119 1492–1514.

[B32] Liebrecht C. Tsaousi C. van Hooijdonk C. (2021). Linguistic elements of conversational human voice in online brand communication: manipulations and perceptions. *J. Bus. Res.* 132 124–135. 10.1016/j.jbusres.2021.03.050

[B33] Lin Y.-C. Wang K.-Y. (2016). Local or global image? The role of consumers’ local–global identity in code-switched ad effectiveness among monolinguals. *J. Advert.* 45 482–497.

[B34] Liu S. Gui D. Y. Zuo Y. Dai Y. (2019). Good slang or bad slang? Embedding internet slang in persuasive advertising. *Front. Psychol.* 10:1251. 10.3389/fpsyg.2019.01251 PMC6566129 31231278

[B35] Luffarelli J. Feiereisen S. Zoghaib A. (2021). More innovative but less socially responsible: the influence of using metaphors in marketing communications on product perception, choice, and adoption intention. *Psychol. Mark.* 38 1–17.

[B36] Luna D. Peracchio L. A. (2005). Sociolinguistic effects on code-switched ads targeting bilingual consumers. *J. Advert.* 34 43–56.

[B37] Mairesse F. Walker M. A. (2011). Controlling user perceptions of linguistic style: trainable generation of personality traits. *Comput. Ling.* 37 455–488.

[B38] Moore E. (2012). The social life of style. *Lang. Lit.* 21 66–83.

[B39] Norton R. W. (1978). Foundation of a communicator style construct. *Hum. Commun. Res.* 4 99–112. 10.1111/j.1468-2958.1978.tb00600.x

[B40] Nunnally J. (1978). *Psychometrical Theory*, 2nd Edn. New York, NY: McGraw-Hill.

[B41] Pennebaker J. W. King L. A. (1999). Linguistic styles: language use as an individual difference”. *J. Pers. Soc. Psychol.* 77 1296–1312. 10.1037/0022-3514.77.6.1296 10626371

[B42] Pennebaker J. W. Boyd R. L. Jordan K. Blackburn K. (2015). *The development and Psychometric Properties of LIWC2015.* Austin, TX: University of Texas at Austin.

[B43] Postmes T. Spears R. Lea M. (2000). The formation of group norms in computer-mediated communication. *Hum. Commun. Res.* 26 341–371. 10.1080/13561820220124175 12028896

[B44] Ranaweera A. T. Martin B. A. Jin H. S. (2021). What you touch, touches you: the influence of haptic attributes on consumer product impressions. *Psychol. Mark.* 38 183–195. 10.1002/mar.21433

[B45] Schank R. C. (1982). *Dynamic Memory: a Theory of Reminding and Learning in Computers and People.* Cambridge: Cambridge University Press.

[B46] Sproull L. Kiesler S. (1986). Reducing social context cues: electronic mail in organizational communication. *Manag. Sci.* 32 1492–1512.

[B47] Sujan M. Bettman J. R. (1989). The effects of brand positioning strategies on consumers’ brand and category perceptions: some insights from schema research. *J. Mark. Res.* 26 454–467. 10.1177/002224378902600407

[B48] Tagliamonte S. A. (2016). So sick or so cool? The language of youth on the internet. *Lang. Soc.* 45 1–32.

[B49] Tarabashkina L. Tarabashkina O. Quester P. Soutar G. N. (2020). Does corporate social responsibility improve brands’ responsible and active personality dimensions? An experimental investigation. *J. Product Brand Manag.* 30 1016–1032.

[B50] Tsalikis J. Ortiz-Buonafina M. LaTour M. S. (1992). The role of accent on the credibility and effectiveness of the international business person: the case of Guatemala. *Int. Mark. Rev.* 9 57–72.

[B51] Turkle S. (1995). *Life on the Screen.* New York, NY: Simon & Schuster.

[B52] Valkenburg P. M. Peter J. (2009). The effects of instant messaging on the quality of adolescents’ existing friendships: a longitudinal study. *J. Commun.* 59, 79–97.

[B53] Valkenburg P. M. Peter J. Walther J. B. (2016). Media effects: theory and research. *Annu. Rev. Psychol.* 67 315–338. 26331344 10.1146/annurev-psych-122414-033608

[B54] Webster C. Sundaram D. S. (2009). Effect of service provider’s communication style on customer satisfaction in professional services setting: the moderating role of criticality and service nature. *J. Serv. Mark.* 23 104–114.

[B55] Wong K. F. Xia Y. Li W. (2006). Linguistic and behavioral studies of Chinese chat language. *Int. J. Comput. Processing Oriental Lang.* 19 133–152. 10.1142/s0219427906001475

